# The interplay between the marine diazotroph *Vibrio diazotrophicus* and its prophage shapes both biofilm structure and nitrogen release

**DOI:** 10.1128/aem.01564-25

**Published:** 2025-12-22

**Authors:** Louise Mahoudeau, Pauline Crétin, Aurélie Joublin-Delavat, Sophie Rodrigues, Clara Guillouche, Isabelle Louvet, Nadège Bienvenu, Claire Geslin, Gabriel Dulaquais, Jean-François Maguer, François Delavat

**Affiliations:** 1 Nantes Université, CNRS, US2B, UMR6286, Nantes, France; 2 Univ Brest, CNRS, IRD, Ifremer, UMR6539, LEMAR, Plouzané, France; 3 Laboratoire de Biotechnologie et Chimie Marines, Université Bretagne Sud, EMR CNRS 6076, IUEM 27100, Lorient, France; 4 Nantes Université, CNRS, UMR6230, CEISAM, Nantes, France; 5 Univ Brest, Ifremer, BEEP, Plouzané, France; Indiana University Bloomington, Bloomington, Indiana, USA

**Keywords:** marine diazotrophs, prophage, biofilm, nutrient cycling, *Vibrio*, nitrogen

## Abstract

Marine environments are frequently oligotrophic, characterized by low amount of bioassimilable nitrogen sources. At the global scale, the microbial fixation of N₂, or diazotrophy, represents the primary source of fixed nitrogen in pelagic marine ecosystems, playing a key role in supporting primary production and driving the export of organic matter to the deep ocean. However, given the high energetic cost of N₂ fixation, the active release of fixed nitrogen by diazotrophs appears counterintuitive, suggesting the existence of alternative passive release pathways that remain understudied to date. Here, we show that the marine non-cyanobacterial diazotroph *Vibrio diazotrophicus* is endowed with a prophage belonging to the *Myoviridae* family, whose expression is induced under anoxic and biofilm-forming conditions. We demonstrate that this prophage can spontaneously excise from the genome of its host and that it forms intact and infective phage particles. Moreover, phage-mediated host cell lysis leads to increased biofilm production compared with a prophage-free derivative mutant and to increased release of dissolved organic carbon and ammonium. Altogether, the results suggest that viruses may play a previously unrecognized role in oceanic ecosystem dynamics by structuring microhabitats suitable for diazotrophy and by contributing to the recycling of (in)organic matter.

**IMPORTANCE:**

Diazotrophs are key players in ocean functioning by providing fixed nitrogen to ecosystems and fueling primary production. However, from a physiological point of view, the active release of nitrogenous compounds by diazotrophs is paradoxical, since they would invest in an energy-intensive process and supply nutrient to non-sibling cells, with the risk of being outcompeted. Therefore, alternative ways leading to the release of fixed nitrogen must exist. Here, we show that the marine non-cyanobacterial diazotroph *Vibrio diazotrophicus* possesses one prophage, whose activation leads to cell death, increased biofilm production, and the release of dissolved organic compounds and ammonium. Taken together, our results provide evidence that marine phage–diazotroph interplay leads to the creation of microhabitats suitable for diazotrophy, such as biofilm, and to nutrient cycling, and contributes to better understanding of the role of viruses in marine ecosystems.

## INTRODUCTION

The process of dinitrogen (N_2_) fixation, or diazotrophy, is restricted to a small part of polyphyletic prokaryotes known as diazotrophs. For decades, free cyanobacteria, particularly those belonging to the genus *Trichodesmium*, are considered to be responsible for much of N_2_ fixation in the oceans (1). However, more recent findings have greatly expanded our understanding of marine N_2_ fixation globally. Indeed, studies based on the amplification of *nifH*—a recognized marker of N_2_ fixation(2, 3)—along with whole genome assembly from Tara Ocean expeditions (4) have shown the widespread occurrence of non-cyanobacterial diazotrophs (NCDs) in the oceans. Remarkably, NCDs were sometimes found in greater abundance than cyanobacteria, accounting for up to 92.6% of the diazotrophs in the collected metagenomes from the 0.8 μm to 2,000 μm size fraction (4). These results suggest that NCDs may play a major role in the marine N_2_ fixation activity. Until now, the strategies deployed by these NCDs during N_2_ fixation remain understudied. We recently demonstrated that the marine diazotroph *Vibrio diazotrophicus* is able to utilize a wide variety of organic and inorganic sources upon shifting to diazotrophic conditions and to hyper-respirate to decrease O_2_ tension. Moreover, we showed that the strain modulates the proportion of nitrogenase-expressing cells during diazotrophic growth in an ammonium-dependent manner, and that this phenotypic heterogeneity might be a conserved trait among marine NCDs (5). In addition, marine NCDs are frequently found in large size fractions of the water column, suggesting cell aggregation and/or colonization of large particles (4). This aggregate/biofilm production may be a strategy to provide low-O_2_ microenvironments required for N_2_ fixation. Supporting this hypothesis, the marine diazotroph *Pseudomonas stutzeri* BAL361 produces cell aggregates under oxic growth with N_2_ as sole nitrogen source (6), and *V. diazotrophicus* NS1 increases biofilm production under soluble reactive nitrogen (SRN)-limited conditions (7).

Irrespective of the strategies deployed by diazotrophs, the diazotrophically derived nitrogen is subsequently assimilated by the N_2_-fixing cell to create new biomass (8). Nevertheless, a fraction of this nitrogen can be released extracellularly, potentially fueling the broader (photoautotrophic) plankton community. This release of organic and/or inorganic nitrogen-containing molecules might involve dedicated transporters. However, because diazotrophs invest energy in N_2_ fixation to (in)directly feed non-sibling cells, with the risk of being outcompeted, an alternative way to release the fixed nitrogen may occur, although this has not been investigated. One such alternative might be viruses, whose lytic activity may play fundamental but still understudied roles in aquatic food webs (9).

In this study, we demonstrate that biofilm production and the release of carbon and nitrogen to the environment by the marine diazotroph *V. diazotrophicus* at least partly rely on the activation of a prophage. We provide evidence that viruses may play a previously unrecognized role in oceanic ecosystem functioning by structuring microhabitats suitable for diazotrophs and by recycling of (in)organic matter.

## RESULTS

### *V. diazotrophicus* engages in an intensive transcriptomic remodeling to anoxia and in biofilm

**Fig 1:**
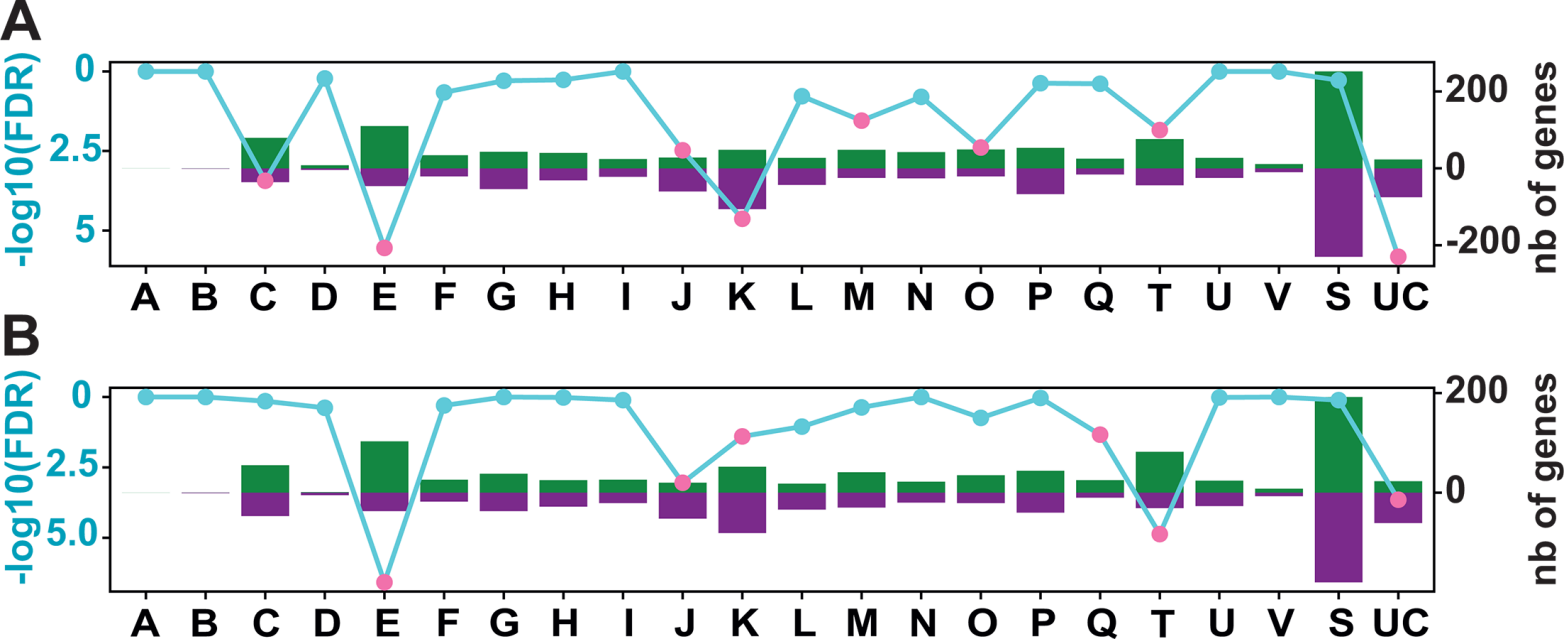
Global transcriptomic response of *V. diazotrophicus* when grown in biofilm (**A**) or in anoxia (**B**) compared to liquid aerobic growth. The vertical bars represent the number of upregulated (green) and downregulated (purple) genes in each COG category. The secondary y-axis (left) shows the statistical significance of the Fisher exact test, represented as -log10(FDR). A threshold of significance (-log10(FDR) ≥ 1.30) is indicated by pink dots, while non-significant values are marked in cyan. COG letters correspond to the following categories: A: RNA processing and modification; C: Energy production and conversion; D: Cell cycle control, cell division, and chromosome distribution; E: Amino acid transport and metabolism; F: Nucleotide transport and metabolism; G: Carbohydrate transport and metabolism; H: Transport and metabolism of coenzymes; I: Lipid transport and metabolism; J: Ribosome translation, structure, and biogenesis; K: Transcription; L: Replication, recombination, and repair; M: Cell wall, membrane, and envelope biogenesis; N: Cell motility; O: Post-translational modification, protein turnover, and chaperones; P: Transport and metabolism of inorganic ions; Q: Biosynthesis, transport, and catabolism of secondary metabolites; S: Function unknown; T: Signal transduction mechanisms; U: Intracellular traffic, secretion, and vesicular transport; UC: Unknown genes; and V: Defense mechanisms.

*V. diazotrophicus* NS1 was recently shown to produce a thicker biofilm under SRN-limiting conditions (7), while the presence of O_2_ is known to inhibit the nitrogenase. To have a global overview of the adaptive response of this facultative anaerobe when grown under anoxic conditions (called “modified diazotrophic medium for *Vibrio*” (MDV) anoxia) or within a biofilm (MDV biofilm), we first aimed to perform a global transcriptomic approach. Each condition was compared to the reference condition in which cells were grown in liquid culture under oxic conditions (MDV), the MDV medium being a SRN-limited medium. A total of 1,540 and 1,883 genes were significantly differentially expressed in the comparisons of MDV vs MDV anoxia and MDV vs MDV biofilm (Fig. 1; Fig. S1; Table S4), corresponding to differential expression of 35–43% of the entire genome (4,431 genes). When comparing MDV and MDV in anoxia, 750 genes (49% of the differentially expressed genes [DEGs]) were significantly upregulated in anoxia, while 790 genes (51% of the DEGs) were downregulated (Tables S1 and S4). Similar proportions were found when comparing MDV with MDV in biofilm, with 910 genes (48% DEGs) being overexpressed in biofilm and 973 (52% DEGs) being underexpressed in biofilm (Tables S1 and S4).

In order to further investigate the metabolic remodeling of this strain under anoxia and biofilm conditions, DEGs were classified by Clusters of Orthologous Genes (COGs; Fig. 1; Fig. S1). In total, between 20.5 and 31.6% of the DEGs were not categorized (either category S “Unknown Function” or UC “Unclassified,” Fig. S1).

We subsequently performed a Fisher’s exact overrepresentation test, which determines significantly enriched categories. Interestingly, genes belonging to the “Unclassified” category, representing 98 and 94 genes in the two sets of conditions (Fig. 1), were significantly enriched, which suggests that many of these unknown genes might be involved in the growth in these particular lifestyles (biofilm and anoxia). The switch from growth under oxic to anoxic conditions (Fig. 1B) revealed that *V. diazotrophicus* genes involved in amino acid transport and metabolism (COG E) and signal transduction mechanisms (COG T) were mostly downregulated. In contrast, category J (translation, ribosomal structure, and biogenesis) was significantly upregulated under anoxia, reflecting intense metabolic rewiring. This activity was also observed when comparing MDV with MDV biofilm (Fig. 1A), with genes from categories J and K also mostly overexpressed. In contrast, genes from cells grown in MDV biofilm and categorized in COG C (energy production and conversion), E (amino acid transport and metabolism), M (cell wall/membrane/envelope biogenesis), O (post-translational modification, protein turnover, chaperones), and T (signal transduction) were mostly downregulated, revealing intense cellular reprogramming during biofilm growth.

A key parameter that differs between aerobic MDV growth and growth under biofilm or anoxic conditions is the O_2_ tension. Consistent with this parameter change, the *bo* cytochrome (*cyoABCD* genes, BBJY01_570181 to 570184 according to Magnifying Genomes [MAGE] nomenclature), which operates at high O_2_ tension, was downregulated in both biofilm and anoxic conditions, while the fumarate reductase (*frdABCD* genes, BBJY01_490101 to 490104) was upregulated under anoxia. This latter system allows anaerobic respiration, suggesting that *V. diazotrophicus* is able to use fumarate as alternative terminal electron acceptor. This is in line with the demonstration that at least some vibrios can use fumarate as electron acceptors (10).

Strikingly, the entire *nif* cluster, composed of four regions and spanning 100 kb of the *V. diazotrophicus* NS1 genome, was downregulated (from 2- to 30-fold) under anoxia and when grown in biofilm (Fig. S2). This includes the *nifHDK* genes encoding the key enzymatic nitrogenase complex and suggests that diazotrophic activity is repressed under such conditions.

### RNA-seq analysis reveals the existence of a prophage region in *V. diazotrophicus*

**Fig 2:**
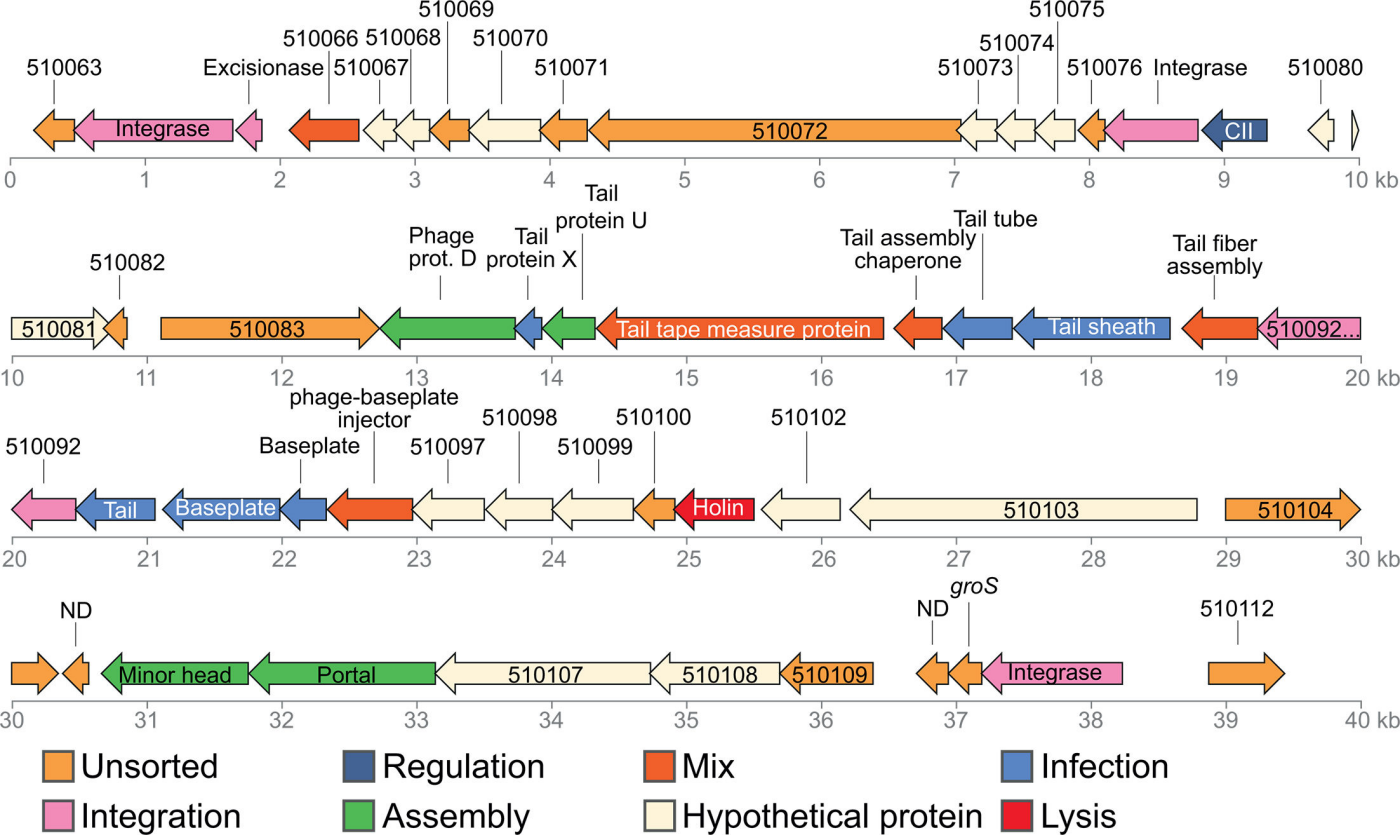
Vdi_1 prophage genes. Every color corresponds to a putative function, according to PhageScope (13) and Virfam (15).

**Fig 3:**
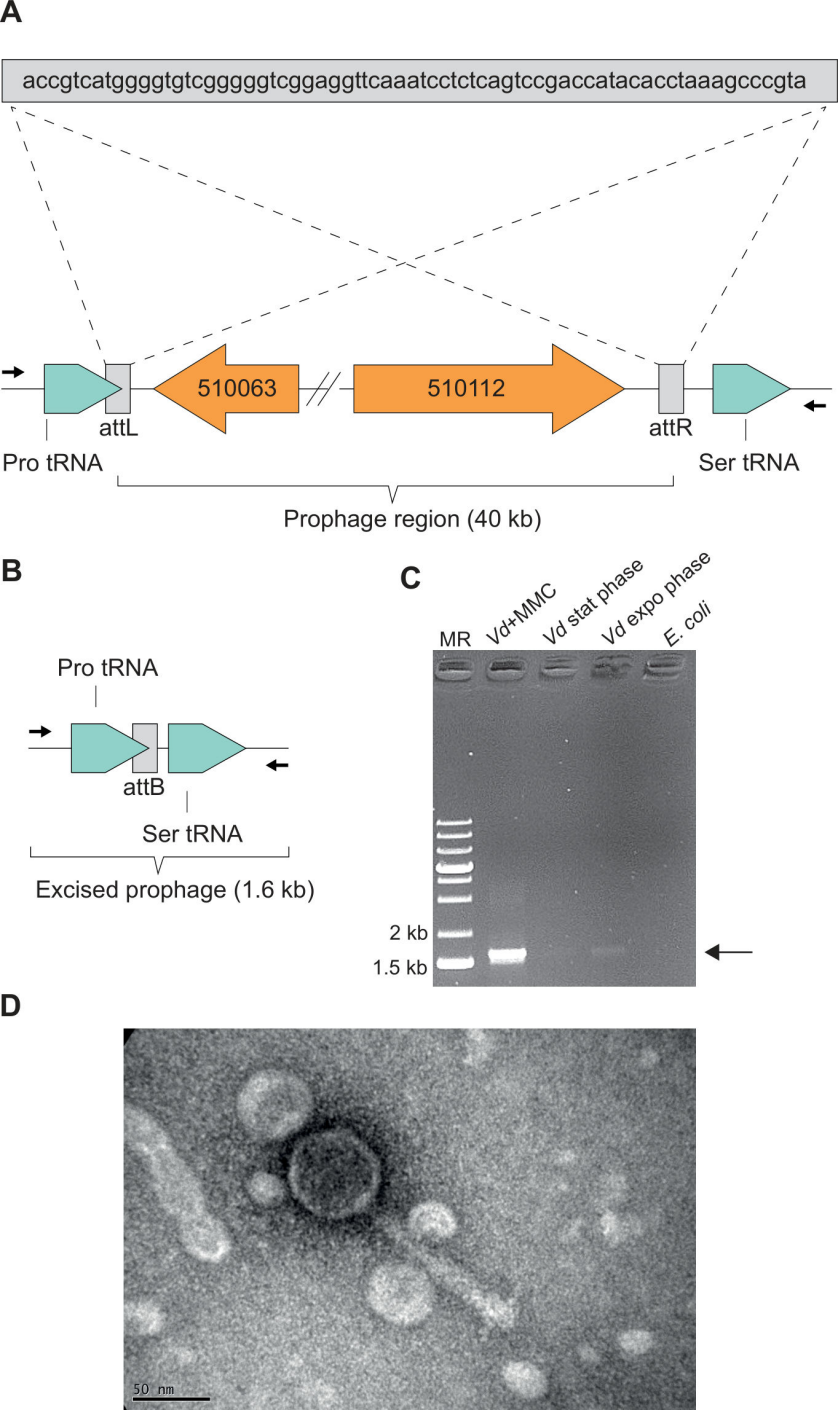
Genomic region of Vdi_1 and prophage excision. (**A**) Localization of the insertion site of Vdi_1. Note the repeated sequences attL and attR flanking Vdi_1 and its insertion at the 3′-end of Pro tRNA. (**B**) Genomic region of *V. diazotrophicus* NS1 upon prophage excision. Arrows represent the primers used to test Vdi_1 excision. (**C**) Electrophoresis gel showing prophage excision, using primers flanking Vdi_1 prophage. MMC: Mitomycin C. (**D**) Transmission electron microscopy picture of Vdi_1 particle, released upon spontaneous excision from *V. diazotrophicus* NS1 grown in LB.

In order to have an overview of genomic regions which revealed similarly regulated (indicating operon organization and/or co-regulation of genes), the list of DEGs was sorted according to their gene order, from gene BBJY01_10001 to BBJY01_570386, according to MAGE nomenclature (https://mage.genoscope.cns.fr/microscope/mage/index.php). This clustering approach led to the discovery of a 40 kb region that revealed similarly overexpressed (from 3- to 40-fold) under both anoxic and biofilm conditions (Table S5 and Fig. S3). This region comprised genes BBJY01_510063 to BBJY01_510112, mostly encoding uncharacterized proteins, but with some genes showing low yet significant similarity to phage genes (Fig. 2). Interestingly, this 40 kb region encompassed a potential prophage region identified by the Phigaro tool of the MAGE platform. Phigaro suggested a 20,396 bp long prophage region composed of 24 genes (BBJ001_510081 to BBJ001_510104, according to MAGE annotation). Given the discovery of a potentially co-regulated 40 kb region by transcriptomic analysis and the fact that this region encompassed a potential prophage region, we hypothesized that the actual prophage region was longer than the one initially discovered by Phigaro and was in fact 40 kb long. This hypothesis was further reinforced by the detection of two tRNA genes flanking this region (Fig. 3A), tRNA genes being known hotspots for phage and other mobile genetic elements insertion (11, 12). Moreover, a perfect 68 bp duplicated sequence flanked this 40 kb region and overlapped with the end of the Pro-tRNA gene sequence (found in the left end of the region, Fig. 3A). This potential 40 kb prophage region was considered as the potential prophage region, and its corresponding DNA sequence was used for further analyses. We subsequently analyzed the potential prophage genome with PhageScope (13). Analysis revealed that the prophage belongs to the *Myoviridae* family, characterized by an icosahedral head and a contractile tail sheath (14). This prophage region comprised the genes encoding an integrase, required for phage DNA site-specific integration; an excisionase required for phage DNA excision; and a CII protein, responsible for the lysogenic/lytic phase switch (Fig. 2). Genes encoding the structure of the viral particle were also present, along with a gene encoding a potential holin required for cell lysis. Taken together, this region comprised all genes required to produce an active prophage which we named Vdi_1.

Importantly, Vdi_1 constitutes the only prophage region detected by Phigaro, suggesting that *V. diazotrophicus* NS1 is endowed with only one prophage. Moreover, Vdi_1 prophage DNA was not detected in the genome of eight other *V. diazotrophicus* strains (strains 60.18M, 65.7M, 60.27F, 60.6F, 65.10M, 99A, 60.6B, and HF9B; data not shown), and its closest relative is a prophage from *Vibrio plantisponsor* LMG24470 (Fig. S4).

### Vdi_1 is an active prophage

We next sought to demonstrate that Vdi_1 is an active prophage. If this hypothesis proves true, the viral DNA should excise via homologous recombination between the *attL* and *attR* sites of the prophage region (Fig. 3A). Viral DNA excision should therefore lead to the removal of the 40 kb prophage region (Fig. 3B). A PCR was conducted, using a primer pair annealing upstream and downstream of the *attL* and *attR* sites, respectively. Successful amplification of a 1.6 kb fragment would demonstrate successful excision of a prophage (Fig. 3B). We used *V. diazotrophicus* genomic DNA as template, extracted from three growing conditions: Lysogeny Broth (LB) in exponential phase, LB in stationary phase, and LB + Mitomycin C (MMC), a known prophage inducer. Indeed, a bright band at 1.6 kb was observed when using the DNA sample from the MMC-induced culture, indicating a massive 40 kb loss under this condition (Fig. 3C). This experiment proved that Vdi_1 prophage is indeed an active prophage, being able to excise from the genome upon induction. Moreover, a faint but visible band was observed when using uninduced DNA samples (both from exponential- and stationary-phase LB-grown cultures), indicating that Vdi_1 was able to activate and excise spontaneously (Fig. 3C). This spontaneous induction also occurs under anoxic conditions when grown in liquid MDV for 48 hours, confirming the increased expression observed by RNA-seq (Fig. S5).

Finally, an LB-grown overnight culture was centrifuged, and the supernatant was ultracentrifuged to concentrate potential viral particles. The resuspended pellet was observed by transmission electron microscopy, which revealed the presence of intact phage particles of the myovirus type, characterized by an icosahedral head and a thick tail with visible tail fibers (Fig. 3D). The presence of phage particles, together with the fact that the Vdi_1 prophage region was the sole region detected in the genome of *V. diazotrophicus* NS1 by Phigaro, formally demonstrated that Vdi_1 is indeed an active prophage.

### Generation of a prophage-free mutant

In order to understand the potential role(s) of Vdi_1 in the physiology of *V. diazotrophicus* NS1, we decided to create a prophage-free deletion mutant of this strain by deleting the entire Vdi_1 40 kb region using a dedicated suicide plasmid. After two recombination steps, PCR amplifications were performed on potential mutants. The first primer pair flanked the entire Vdi_1 region and gave a bright 1.6 kb band in uninduced cells, revealing the 40 kb deletion. However, since spontaneous excision can occur (Fig. 3C), we used a second primer pair, with primers amplifying a fragment within the Vdi_1 region. The presence of an amplification band in the wild type and its absence in the mutant confirmed that a Vdi_1 deletion mutant was obtained. Finally, we were unable to observe any phage particles in the supernatant of the mutant, unlike the one of the wild type (data not shown), supporting that Vdi_1 was the only prophage of *V. diazotrophicus* NS1 and that the Vdi_1-deletion mutant corresponded to a prophage-free mutant.

### A prophage-free *V. diazotrophicus* mutant is more resistant to mitomycin induction

**Fig 4:**
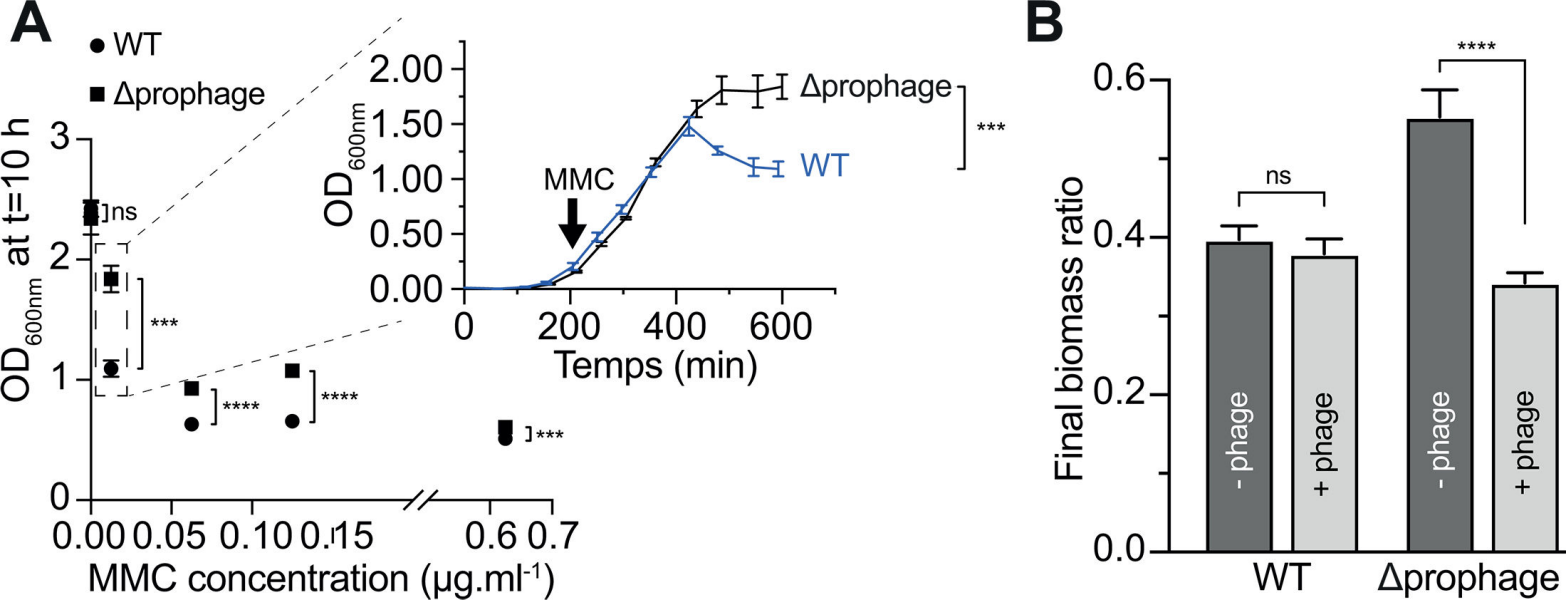
Prophage activation and infection experiments. Sensitivity of *V. diazotrophicus* NS1 and the prophage-free mutant to MMC (**A**). Cultures of both strains were inoculated with MMC at increasing concentrations, and the OD was_600nm_ measured after 10 h. An unpaired two-tailed *t*-test for every MMC concentration was performed. ns, not significant. Inset: The entire growth curves for both strains when grown in LB supplemented with 0.0125 µg/mL MMC. Infection experiment using Vdi_1 and *V. diazotrophicus* NS1 or its prophage-free derivative as preys (**B**). Vdi_1 particles (for the “+Phage” histograms) and the supernatant of the prophage-free mutant (“−Phage” histograms) were used to inoculate either *V. diazotrophicus* NS1 or the prophage-free mutant as prey. Cultures were incubated for 27 h, and the final OD_600nm_ was measured. The y-axis corresponds to the ratio of the final OD_600nm_ obtained in the test experiment by the final OD_600nm_ obtained when adding the TPV-1 buffer. An unpaired two-tailed *t*-test was done to compare the two conditions. ns: not significant. ***: *P* < 0.001. ****: *P* < 0.0001, ns, not significant.

In a second set of experiments, *V. diazotrophicus* NS1 and its prophage-free derivative mutant were grown in LB under oxic conditions. Mitomycin C (MMC) was spiked at different concentrations, and OD_600nm_ was monitored regularly over a 10-hour period to track biomass concentrations. A drastic decrease in OD was observed for *V. diazotrophicus* NS1 with increasing MMC concentrations, dropping from OD 2.4 without MMC to OD 0.5 at 0.625 µg/mL MMC (Fig. 4A). A similar trend was observed for the prophage-free *V. diazotrophicus* mutant, confirming that MMC is a potent anti-microbial (16). Interestingly, at intermediate MMC concentrations, the decrease in final biomass concentration (measured as OD600_nm_) largely differed between the wild type and the mutant, with a significantly greater decrease observed for the wild type. Indeed, growth curves largely overlap between both strains and start to diverge around 200 min post-MMC spiking (Fig. 4, inset). This difference is likely due to the induction of Vdi_1 in the wild-type strain, causing cell lysis and a faster decrease in OD. Supporting this hypothesis, time-lapse movies acquired upon MMC induction at 0.0125 µg/mL showed frequent and rapid cell elongation with aberrant cell shape in wild-type cells, an effect not observed in the prophage-free mutant (Videos S1 and S2).

### Vdi_1 produces infective particles

We then tested whether Vdi_1 particles are able to infect *V. diazotrophicus* cells. Phage particles were collected and used to infect either *V. diazotrophicus* NS1 or the prophage-free mutant. The mutant was included as prey because the prophage located in the genome of the wild type might confer immunity to superinfection by the same virus. However, despite repeated attempts under multiple conditions (+/− O_2_, in LB or MDV media, and using either non-induced or MMC-induced phage-producing cells; see Material and Methods), we were unable to obtain lysis plaques on plates, using either *V. diazotrophicus* or its prophage-free derivative mutant as prey (data not shown). We subsequently measured the growth of both strains, either spiked with the phage suspension or with the suspension from the prophage-free mutant. The results showed that the presence of phage particles induced a significant decrease (*P* < 0.0001) in the biomass of the prophage-free mutant as prey (Fig. 4B). This shows that, despite the absence of visible plaque lysis, Vdi_1 was able to infect and kill prophage-free mutant cells, leading to decreased final biomass.

### Vdi_1 does not play a role in N_2_ fixation under micro-oxic conditions

In order to determine the role Vdi_1 might have in *V. diazotrophicus*, we first sought to determine the impact of Vdi_1 deletion in the physiology of this strain. Our results first demonstrated that the deletion of the Vdi_1 prophage region did not affect the growth of *V. diazotrophicus* when grown in liquid LB, reinforcing the fact that Vdi_1 was generally silent in this strain or unable to infect the wild-type, prophage-containing host (Fig. S6A).

Moreover, when both the wild type and the prophage-free mutant were grown in soft-gellan medium, the strain in which the Vdi_1 region was deleted was still able to grow in the absence of nitrogen sources other than N_2_ and to fix N_2_ at a comparable rate to that of the wild type (Fig. S6B and C). Therefore, the prophage has no role in the diazotrophic activity of *V. diazotrophicus* NS1 when grown under micro-oxic conditions.

### Vdi_1 prophage participates in biofilm production in *V. diazotrophicus*

**Fig 5:**
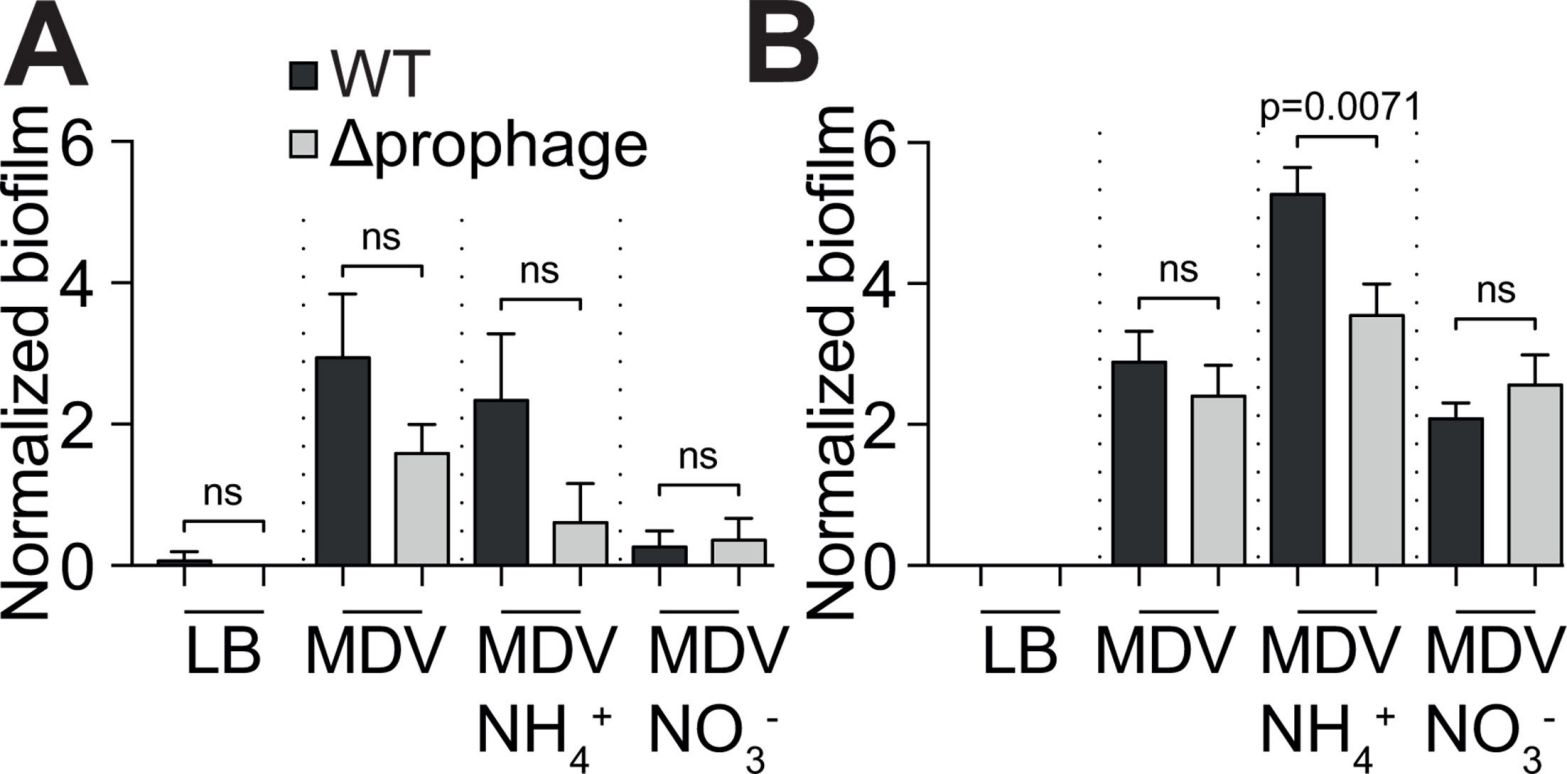
Biofilm production by *V. diazotrophicus* NS1 or the prophage-free mutant under anoxic conditions. Both strains were grown for 24 h (**A**) or 48 h (**B**) in microplates under anoxic conditions, in different media. Biofilm production was normalized by dividing OD_550nm_ by the OD_600nm_ obtained after 24 h (**A**) or 48 h (**B**). Unpaired two-tailed *t*-tests were performed. ns, not significant.

We recently demonstrated that *V. diazotrophicus* NS1 produces a thick biofilm and hypothesized that this biofilm production is an ecophysiological adaptation of this strain to limit O_2_ diffusion with the biofilm, creating micro-oxic microenvironments suitable for diazotrophy (7). As biofilm matrices are known to be composed of polysaccharides, as well as DNA, RNA, and proteins, we hypothesized that Vdi_1-induced cell lysis leads to the release of these organic molecules, which participate in biofilm structuration. To test this hypothesis, we grew *V. diazotrophicus* NS1 and its derivative prophage-free mutant in microplates without shaking, using different growth media, under both oxic and anoxic conditions, and assessed biofilm production after 24 and 48 h of growth. Under oxic conditions, no significant difference was observed between both strains when grown both under static condition (in microplate) and in milli-fluidic using confocal laser scanning microscopy (Fig. S7). In contrast, under anoxic conditions, the prophage-free mutant tended to produce less biofilm than the wild type, a trend that is statistically significant when supplemented with NH_4_^+^ (Fig. 5). These results highlighted that Vdi_1 may participate in biofilm production in anoxic environments, likely through phage activation and cell lysis.

### Vdi_1 prophage activation leads to the release of carbon and nitrogen compounds

**Fig 6:**
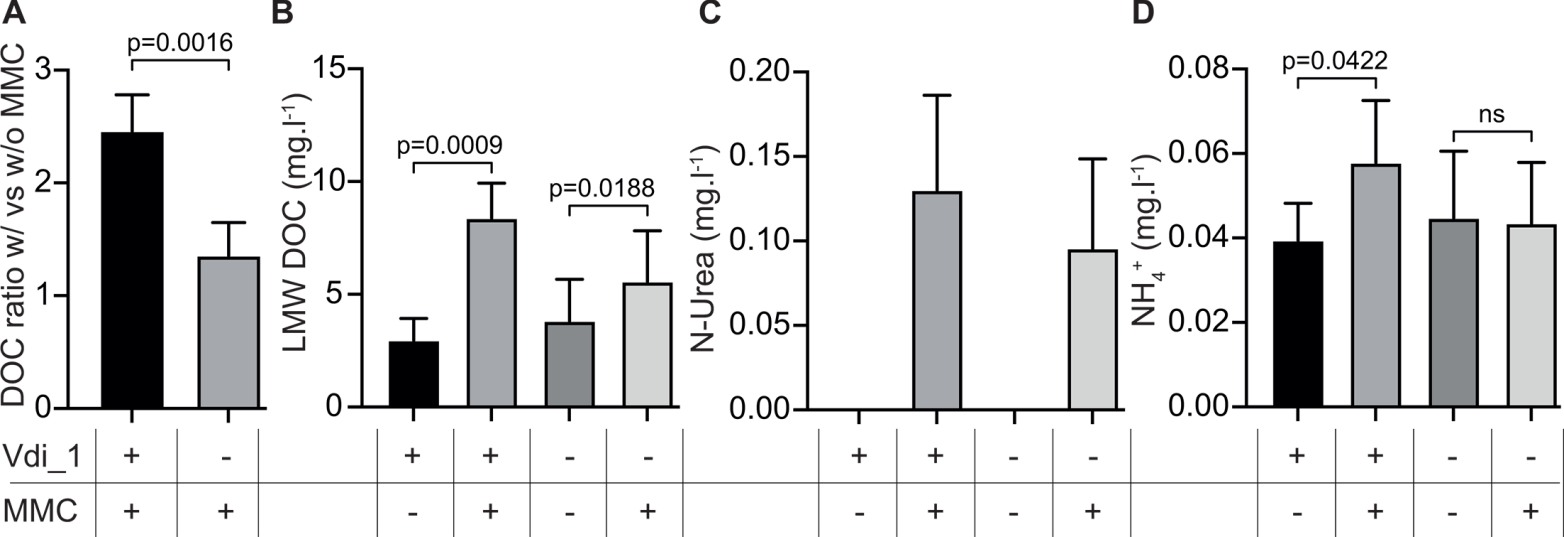
Carbon and nitrogen released by *V. diazotrophicus* and the prophage-free mutant. Total DOC amount released upon MMC induction divided by the total DOC amount released without induction. An unpaired two-tailed Welch’s *t*-test was performed (**A**). Low molecular weight (LMW) (**B**), N-urea (**C**), and NH_4_^+^ (**D**) released by both strains without and with MMC induction. Paired two-tailed *t*-tests were performed for (**B–D**). ns: not significant.

As prophage induction leads to host cell death (Fig. 4), we hypothesized a release of C and N in the external environment during the phage-mediated cell lysis. To test this hypothesis, we first induced both the wild-type strain and the prophage-free mutant with MMC. Then, the amount and nature of C and N compounds in the filtrates were quantified and compared with non-induced samples. Dissolved organic carbon (DOC) was detected in MMC-free samples for both the wild type and the prophage-free mutant (4.5 to 5.8 mg/L from 2 × 10^9^ cells, Table S6). MMC induction led to a 2.5-fold increase in DOC release in the wild-type strain, but only to a 1.4-fold increase in the prophage-free mutant; this difference was significant (Fig. 6A). This increased release of DOC was mostly due to the increased release of low molecular weight (LMW) DOC (Fig. 6B; Table S6), while hydrophobic DOC was released irrespective of MMC induction, and high molecular weight (HMG) and humic DOC were only very modestly detected (Table S6). Strikingly, MMC induction also led to the release of urea in both strains, a molecule that was completely absent in uninduced ones (Fig. 6C). Moreover, ammonium releases significantly (*P* < 0.05) increased after induction in the wild-type samples but not in the prophage-free mutant (Fig. 6D).

## DISCUSSION

Viruses are the most abundant entities on earth and have long been viewed as dangerous entities, causing death and destruction at their vicinity. As a consequence, viruses play a fundamental ecological role by controlling the number of hosts. In addition, virus-mediated cell lysis can lead to the release of (i) (in)organic matter participating in nutrient recycling and (ii) particulate organic matter, which can serve new microenvironments. However, the potential ecological roles of viruses remain largely unexplored and are therefore frequently overlooked in environmental studies. In this study, we provide evidence that a prophage of the marine diazotroph *V. diazotrophicus* NS1 plays a role in biofilm structuration and in the release of C and N, demonstrating that viruses can be key players in biogeochemical cycles of these two elements.

In recent years, marine NCDs have been detected associated with animals (17, 18) or in low-O_2_ environments (e.g., in oxygen minimum zones [19]), supporting the idea that hypoxic or anoxic environments are conducive to nitrogenase functioning. In addition, their frequent presence within large particles suspended in the water column (4, 20) suggests a tendency to aggregate and produce biofilm as a strategy to limit O_2_ diffusion. Indeed, sinking particles, including marine snow and fecal pellets, contain a high C:N ratio exceeding the Redfield ratio and can be considered as hotspots for marine NCDs (21). Our results show that *V. diazotrophicus* NS1 engages in massive gene transcription reprogramming upon growth in both anoxia and in biofilm (Fig. 1; Fig. S1), highlighting that these environmental shifts correspond to major lifestyle changes for this strain.

Strikingly, the entire *nif* cluster was downregulated under these conditions (Fig. S2). The down-expression of the nitrogenase is consistent with the inability of *V. diazotrophicus* NS1 to fix N_2_ under anoxic conditions (5), while micro-oxic conditions have been shown to be conducive to diazotrophy (7). Therefore, the observed decreased nitrogenase expression in anoxia shows that a low—but not null—O_2_ tension is necessary for diazotrophy to occur, likely to produce ATP by respiration, which in turn fuels the nitrogenase activity. Enhanced nitrogenase activity under low O_2_ levels has been observed in some facultative anaerobe NCDs (6), while anoxia can also sustain diazotrophy in other NCDs (22, 23), highlighting the existence of various energetic strategies to fuel diazotrophy. The downregulation of the *nif* cluster under biofilm conditions is also unexpected and contradicts our previously published results highlighting an increased biofilm production under SRN-limited conditions (7). This discrepancy may be due to the timing of RNA extraction for the RNA-seq analysis, which provides only a snapshot image of possible successive events occurring during the time course of biofilm production.

Strikingly, growth in biofilm and anoxia induced a prophage in *V. diazotrophicus* NS1, which was shown to be infectious (Fig. 3 to 5). Prophage activation typically occurs when host cells experience physiological stress, such as low energetic status or oxidative stress, enabling the viruses to escape a possibly dying cell to find a new host (24). This suggests that such conditions trigger a stress to *V. diazotrophicus* NS1, probably linked to the energetic status of the host. This hypothesis is further supported by the overexpression of stress response genes such as *ibpA* (BBJY01_360039, 40- to 47-fold in anoxia and biofilm, respectively, Table S5), encoding a small heat-shock protein whose importance in biofilm formation has been demonstrated in *Vibrio fischeri* (25), and the CpxARP envelop stress system (BBJY01_490067 to 490069, induced up to 890-fold in anoxia, Table S4), which is implicated in biofilm formation and resistance to stress in *Vibrio alginolyticus* (26).

In line with our results, numerous studies have demonstrated prophage activation upon bacterial growth in biofilm (27), and this activation may play a role in biofilm structuration (28, 29). Indeed, a bacterial biofilm is a complex structure composed of polysaccharides, but also of DNA, RNA, and proteins (30). Phage-mediated biofilm production can affect downstream processes, as shown in *Pseudomonas aeruginosa* carrying the prophage Pf4, which participates in the virulence of this strain (31), while a decreased virulence was observed in a phi458-deleted mutant strain of *E. coli* (32). As biofilm seems to be the preferred lifestyle of marine NCDs (4, 20), we suggest that prophage activation may regulate diazotrophy and, therefore, oceanic biogeochemical cycles by participating in biofilm structuration. On the other hand, we also showed that Vdi_1 has no role in nitrogenase activity when grown micro-oxically in soft-gellan (Fig. S6). However, we recently demonstrated that, in this experimental context, cells are free-living and do not form aggregates (7). Whether Vdi_1 indirectly affects diazotrophy when cells switch to multicellularity by biofilm production (i.e., when grown oxically under static condition) remains to be demonstrated.

To our knowledge, Vdi_1 represents the first member of demonstrated active prophages within marine NCDs. In the archaeon *Methanothermococcus thermolithotrophicus*, putative viral genes were shown to be overexpressed when grown under diazotrophic conditions, but no complete prophage was detected (23). As their activation may provide fixed nitrogen to marine microbial communities, viruses that infect them NCDs likely represent a significant yet still underexplored component of marine ecosystem dynamics (33). We showed here that prophage-mediated host cell lysis triggers the release of significant amounts of LMW-DOC (Fig. 6A) and ammonium. Therefore, it is tempting to speculate that *V. diazotrophicus* cells in poor condition (e.g., because of low energetic status or oxidative stress) may undergo Vdi_1-induced cell lysis, leading to the release of SRN and carbon sources to help clonemate survive in harsh conditions through a process known as kin selection (34). Moreover, from an ecological point of view, the release of such molecules from diazotrophs may play a crucial role in ecosystem functioning, as the diazotrophically derived nitrogen can be used by the remaining (phyto-)planktonic community, fueling primary production. Interestingly, a recent RNA-seq approach on *V. diazotrophicus* NS1 grown under diazotrophic conditions showed the strain’s capacity to assimilate both ammonium and urea, two N sources preferentially taken up by planktonic communities (5).

To our knowledge, the only report showing the role of a prophage in the release of fixed nitrogen came from *Trichodesmium* (35). Here, we demonstrated that Vdi_1-mediated cell lysis leads to the release of 6 µmol of DOC and 16.3 µmol of NH₄^+^ from 2 × 10^9^ cells (after subtracting the amount of DOC or NH_4_^+^ released by the prophage-free mutant upon MMC induction). This corresponds to a release of 3 fmol DOC and 8.15 fmol NH_4_^+^ per *V. diazotrophicus* cell. Assuming an abundance of 10^5^ single-cell free-living NCDs per liter in surface waters (36) and given that most bacteria are endowed with at least one prophage, this corresponds to a potential phage-induced release of 300 pmol DOC and 815 pmol NH_4_^+^ per liter of seawater by NCDs. This potential release is highly conservative, as NCDs may grow at higher density when associated with aggregates and in aphotic waters or deep-sea sediments. Therefore, given the high prevalence of marine NCDs in ocean (4, 37, 38), phage-mediated host killing has the potential to significantly contribute to oceanic nitrogen and carbon cycle, especially in oligotrophic tropical and subtropical waters.

In conclusion, this study unveils the existence of a prophage in the marine NCD *V. diazotrophicus*. Its activation leads to biofilm structuration, which is known to be hotspots for diazotrophy and to diazotroph-host-cell lysis, leading to the release of SRN and carbon compounds into the environment. Given the prevalence of proviruses in bacterial and archaeal genomes including diazotrophs, our study provides evidence that proviruses may largely contribute to biogeochemical cycles and oceanic ecosystem functioning.

## MATERIALS AND METHODS

### Strains and culture conditions

The *V. diazotrophicus* strain used in this study was *V. diazotrophicus* NBRC 103148 (also named NS1). *V. diazotrophicus* and *Escherichia coli* strains were routinely grown in Lysogeny Broth (LB Lennox) at 30°C and 37°C, respectively. When required, *V. diazotrophicus* was grown in SRN-deficient media (MDV [5]). If necessary, the following compounds were added to the media: either before autoclaving for NH_4_Cl (1 g/L), sodium thioglycolate (ref. T0632, Sigma, 76 mg/L), resazurin (R7017, Sigma-Aldrich) (500 μg/L from a 500 mg/L stock solution), and agar (1.5%), or after autoclaving for cysteine-HCl (ref. 23255.186, VWR, 0.05% from a 5% filtered-sterilized solution in MQ water), NaNO_3_ (46.67 mg/L from a 46.67 g/L autoclaved stock solution), trimethoprim (Trim, 10 μg/mL), chloramphenicol (Cm, 5 μg/mL), glucose (Glc, 0.3 g/L), L-arabinose (L-ara, 0.2%), mitomycin C (MMC, ref. 51854, Ozyme) (prepared at 5 mg/mL in DMSO and diluted at 5 to 500 μg/mL stock solutions in MQ water, used at concentrations between 0.001 and 1 μg/mL), and D-aminopimelic acid (DAP, 0.3 mM).

Doubling times were determined by spectrophotometry by inoculating (in triplicate) overnight LB-grown cultures into 100 mL Erlenmeyer flasks containing 20 mL of LB with shaking, and by regularly measuring OD_600nm_. The doubling time was measured from the exponential growth phase of each culture.

Strains, plasmids, and primers used in this study are summarized in Tables S1 to S3.

### Strain constructions and DNA techniques

Standard procedures were used for all molecular experiments, following the suppliers’ recommendations.

Prophage deletion was performed in *V. diazotrophicus* using an already established protocol (7, 39). Briefly, fragments of approximately 800 bp flanking the prophage region were PCR-amplified, fused together, and cloned into the suicide plasmid pLP12 (40) using *Eco*RI and *Xma*I. The resulting pFD156 plasmid was introduced in *E. coli* β3914 (41), which served as a donor during bacterial conjugation with *V. diazotrophicus*. After plating on LB + Cm + Glc, obtained colonies in which the pFD156 plasmid had integrated into the genome of *V. diazotrophicus* were grown overnight in LB + L-arabinose, followed by plating on LB + L-arabinose. Deletion of the prophage was verified by colony PCR using a primer pair flanking the prophage region (primers 240,217 and 240,218) and an internal primer pair (primers 240,228 and 240,230). Strain *V. diazotrophicus*, in which the prophage has been deleted, was stored at −80°C (strain 588). The prophage-free mutant, as well as *V. diazotrophicus* wild type, was stored at UBOCC culture collection under the numbers UBOCC-M-3575 and UBOCC-M-3576, respectively.

The *in silico* plasmid map and DNA sequences are available upon request.

### Soft-gellan assay

The ability of *V. diazotrophicus* and the prophage-free mutant to grow under SRN-free conditions was assessed using a recently described soft-gellan assay (7), with the following modifications to obtain cleaner bands and results: instead of using an overnight culture of *V. diazotrophicus* strains, overnight cultures were back diluted in fresh LB, allowed to grow until OD_600nm_ reached around 0.2–0.5, and 1 mL of these cultures was washed three times with MDV without yeast extract and adjusted to OD_600nm_ 0.5. Fifty microliters of these washed cultures was used to inoculate soft-gellan tubes. Growth was demonstrated by the presence of a clear growing ring in the tube after 3 to 4 days of growth.

### Acetylene reduction assay

Strains were grown in soft gellan as described above, except that 100 μL of overnight culture, washed three times and resuspended in half the initial volume, were inoculated. Tubes were sealed with rubber stoppers with reversible edges (ref. ZZ124591, Sigma-Aldrich). A 1 mL gas-tight syringe (Hamilton) was used to remove 900 μL of the headspace of the tube. Subsequently, 900 μL of acetylene was injected. Tubes were incubated at 20°C without shaking. Acetylene reduction to ethylene by the nitrogenase was measured at day 3 and day 6 by gas chromatography (column: GS-Alumina, 1153552, Agilent; 50 m × 0.53 mm, 0.25 μm; system: HP 6890 Series, Agilent). At each sampling point, the population-wide ethylene to acetylene ratio was quantified. Non-inoculated soft-gellan tubes served as negative controls, where only acetylene was detected.

### Biofilm production in microplates

The propensity of *V. diazotrophicus* and the prophage-free mutant to produce biofilm was quantified under various conditions using crystal violet, as recently presented (7). Briefly, *V. diazotrophicus* or the prophage-free mutant was grown overnight in LB, before being washed three times, either with fresh LB or with MDV. Cultures were adjusted to an OD_600nm_ of 0.01 in LB, MDV, MDV + NO_3_^−^, or MDV + NH_4_Cl. Two hundred microliters of these OD-adjusted cultures was inoculated in quadruplicate in polystyrene 96-well microplates (ref. 330035, Dutscher). Microplates were placed for 24 or 48 h at 30°C without shaking. At the end of the incubation, OD_600nm_ was measured in a microplate reader (TECAN Infinite M1000) to assess cell growth. Cell suspensions were removed by inversion, and 220 μL of crystal violet (0.1%) was added into the wells, followed by a 10-minute incubation. After two rinsing steps (by plunging the microplate in a distilled water bath), microplates were dried for 24–48 h at room temperature, upside down. Crystal violet was subsequently dissolved with 30% acetic acid for 10 min before OD_550nm_ measurement. Biofilm production was corrected by dividing the OD_550nm_ measured by the OD_600nm_ corresponding to cell growth after 24 or 48 h.

For the quantification of biofilm production under anoxic condition, inoculated microplates were placed in GazPak EZ anaerobe pouch system (ref. 260683, Grosseron) and processed as described above.

### Biofilm culture

*V. diazotrophicus* biofilms were grown at 30°C under hydrodynamic conditions in a three-channel flow cell (1 × 4 × 44 mm; Biocentrum, DTU, Denmark) (42). The flow system was assembled, prepared, and sterilized as described by (43). The substratum consisted of a microscope glass coverslip (24 × 50 mm; Knittel Glasser, Braunschweig, Germany). Each channel was inoculated with 250 µL of an overnight culture of *V. diazotrophicus* diluted to an OD_600_ of 0.1 in MD medium. A 2-hour attachment step was performed without any flow of medium. Then, a 5 mL/h flow of MDV medium was applied for 24 h using a Watson Marlow 205U peristaltic pump (Watson Marlow, Falmouth, UK).

Biofilms formed by *V. diazotrophicus* carrying the pFD086 were observed by monitoring the GFP fluorescence with a LSM 900 Confocal Laser Scanning Microscope (Zeiss, Oberkochen, Germany) using a 40× oil immersion objective. GFP was excited at 488 nm, and fluorescence emission was detected between 500 and 550 nm. Images were acquired at intervals of 1 nm throughout the whole depth of the biofilm. ZEN 2.1 software (Zeiss, Oberkochen, Germany) and ImarisViewer (https://imaris.oxinst.com/imaris-viewer) were used for visualization and image processing. Quantitative analyses of image stacks were performed using COMSTAT software (http://www.imageanalysis.dk/) (44). At least three image stacks from each of three independent experiments were analyzed.

### RNA extraction

#### Liquid MDV

Overnight LB-grown preculture was washed three times with MDV without yeast extract. Two hundred microliters of the washed preculture was inoculated into Erlenmeyer flasks (in quadruplicates) with 20 mL MDV and incubated with shaking (200 rpm) at 30°C for 24 h. Samples (5 × 10^8^ cells) were processed using the Direct-zol DNA/RNA miniprep kit (ref. R2080, Zymo Research).

#### MDV under anaerobic conditions

Overnight LB-grown preculture was washed three times with MDV without yeast extract. Two hundred microliters of the washed preculture was inoculated (in quadruplicates) into 100 mL vials containing 100 mL of medium (ref. 33110, Sigma-Aldrich), sealed with a blue rubber stopper (ref. 2048-11800, Bellco Glass). O_2_ was removed by adding sodium thioglycolate in the solution 2 of the MDV medium before autoclaving, by adding 1 mL cysteine-HCl 5% in the reconstituted MDV medium after autoclaving, and by flushing the headspace for 2 min with N_2_ gas immediately after autoclaving, when the temperature was above 70°C (45). The absence of O_2_ was observed the day after, thanks to the addition of resazurin in solution 2 before autoclaving. Incubation was done for 24 h at 30°C with shaking. Samples (5 × 10^8^ cells) were processed using the Direct-zol DNA/RNA miniprep kit.

#### MDV biofilm

Overnight LB-grown preculture was washed three times with MDV without yeast extract. Two milliliters of this washed preculture was adjusted to an OD_600nm_ of 0.01 with MDV with yeast extract and placed in a 24-well polystyrene microplate (ref. CC77672-7524, Starlab). The microplate was incubated for 48 h at 30°C without shaking. Cell suspension was removed by inversion. One hundred fifty microliters of Tri Reagent (ref. 2050-1-50, Zymo Research) was subsequently added and left for 1 h at room temperature. To have enough material, two wells were pooled to form one replicate, and each quadruplicate was centrifuged at 12,000 × *g* for 1 min, before transferring the supernatant in a RNAse- and DNase-free Eppendorf tube. Samples were processed using the Direct-zol DNA/RNA miniprep kit, starting from step 2.

Every extracted RNA was deposited on gel and stored at −80°C until processing.

### RNA sequencing and data analysis

Total RNA was sent to Eurofins (Germany). The company did the ribosomal RNA depletion, DNase treatment, cDNA library preparation, and Illumina sequencing (NovaSeq 6000 S4 PE150 XP). Sequencing was performed on three replicates per condition (the replicate with the lowest RIN (RNA Integrity Number) according to Eurofins standards was removed).

Initial bioinformatic analysis was performed by Eurofins: 22 millions of raw reads were obtained per sample, which were processed using RiboDetector (46) to remove reads classified as rRNA. High-quality reads were obtained using fastp (47). At least 20.09 millions of “High Quality” reads per sample were obtained. Those reads were aligned to the reference genome of *V. diazotrophicus* (GCA_000740015.1) using STAR (48). Genome-wide quantification was achieved using featureCounts (49). Differential gene expression between two conditions was performed using R/Bioconductor package edgeR (50).

DEGs were initially determined by Eurofins with a cutoff of 0.1 for adjusted *P*-values. We subsequently trimmed the results to retain DEGs having an adjusted *P*-value below 0.05, which was further corrected for false discovery rate (FDR) using the Benjamini-Hochberg method (Table S4).

Figures presenting RNA-seq data were obtained using an in-house written Python script.

### Prophage bioinformatic analysis

The initial screening for phage detection was performed on the MicroScope platform (https://mage.genoscope.cns.fr/microscope/home/index.php) (51) using the Phigaro tool. This region was enlarged to a 40 kb region (see Results) to consider it as a potential prophage region, and the corresponding sequence was used for further analyses. Prophage genome analysis was performed with PhageScope (13), and Virfam (15) was used to classify the phage.

### Electronic microscopy

Precultures of *V. diazotrophicus* and the prophage-free mutant were inoculated in LB. These overnight cultures were used to inoculate 50 mL LB, which were incubated overnight at 30°C. Cultures were subsequently centrifuged at 8,000 rpm for 15 min to remove bacterial cells. Supernatants were ultracentrifuged (Beckman Optima LE-80 K, 45Ti rotor) at 41,000 rpm for 90 min at 10°C. Pellets were resuspended with 150 µL of a dedicated viral TPV1 buffer (Tris-HCl 10 mM, NaCl 100 mM, CaCl_2_ 5 mM). Five microliters was deposited on a TEM grid, followed by the addition of 5 µL of 2% uranyl acetate before microscopic observation.

### Plaque assay

Phage particles from uninduced cultures were obtained as follows: a *V. diazotrophicus* 8-hour preculture was inoculated into 100 mL of LB in a 500 mL Erlenmeyer flask, which was allowed to grow overnight before phage concentration by ultracentrifugation at 41,000 rpm during 90 min at 10°C (Beckman Coulter XE-90 ultracentrifuge, rotor: SW41-Ti, tube: 13.2 mL Ultra-Clear). The supernatant was removed, and the viral pellet was resuspended in 1 mL of viral buffer.

Phage particles from induced cultures were obtained by inoculating an overnight preculture in 100 mL of LB. When OD_600nm_ reached 0.2, mitomycin C (MMC, 0.0125 or 0.125 µg/mL) was added 4 h later, and phage particles were collected as described above.

One hundred microliters of the viral suspension (undiluted or diluted 1 times) was mixed with 100 µL of MDV-washed prey (either *V. diazotrophicus* or the prophage-free mutant, adjusted at OD_600nm_ = 1 from a stationary-phase culture) and incubated for 30 min at 30°C. Subsequently, the mixture was used to inoculate 10 mL of an agar-top medium containing agar (7 g/L) with LB (20 g/L) supplemented with CaCl_2_·2H_2_O (0.37 g/L final concentration from a 147.01 g/L stock solution), MgCl_2_ (0.24 g/L final concentration from a 95.21 g/L stock solution), before being poured on top of LB plates. When working under anoxic conditions, MMC (0.0125 µg/mL) was added in the agar top in which arabinose (2 g/L^−^) was also added. Anoxia was obtained by placing plates in a GazPack.

Plaque assays were also tested in MDV under anoxic conditions, during which MDV (instead of LB) was used in the agar-top medium, and the mixture was poured onto MDV plates, incubated in GazPacks.

The same experiments were performed in parallel with the supernatant of a prophage-free derivative mutant, which served as a negative control (no phage production).

For every condition, plates were incubated for 24 h and 48 h at 30°C before lecture.

### Infection assay in microplates

*V. diazotrophicus* and the prophage-free mutant were grown in LB, and phage suspension (or lack of it for the prophage-free mutant) was obtained as described for the plaque assays. In parallel, overnight cultures of both strains were washed twice and diluted to an OD_600nm_ of 0.01. One hundred eighty microliters was deposited into polystyrene 96-well microplates and incubated in TECAN microplate reader at 30°C, and OD_600nm_ was measured every 30 min. After 3 h of growth, 20 µL of either viral buffer, phage suspension (coming from the wild-type strain), or phage-free suspension (coming from the prophage-free mutant) were added to the wells, and OD_600nm_ was measured every 30 min for 24 h. The final OD_600nm_ of each well (from the wild-type strain or the mutant, in contact with either the phage suspension or the phage-free suspension) was divided by the mean of the final OD_600nm_ obtained from the corresponding inoculations spiked with the TPV-1 buffer.

### Timelapse microscopy

Overnight precultures of *V. diazotrophicus* or the prophage-free mutant were used to inoculate fresh LB. After 3 h of re-growth, cells were deposited onto a 1% agarose patch containing MDV + MMC (0.0125 µg/mL). Images of the same field were taken every 30 min for 5 h, using a Eclipse Ni-E microscope (Nikon) equipped with a 100× Apochromat oil objective.

### Quantification of carbon and nitrogen released

Both *V. diazotrophicus* and the prophage-free mutant were grown overnight in LB. The next morning, cultures were adjusted to an OD_600nm_ of 0.01 in Erlenmeyer flasks containing 30 mL LB, in quintuplicate. After around 5 h, OD_600nm_ was measured for each culture. Cells were then washed twice with 15 mL NaCl (35 g/L) and resuspended in 1 mL NaCl. Each cell suspension was used to inoculate two Erlenmeyer flasks (per suspension), each containing 15 mL NaCl (35 g/L) to achieve a final concentration of 2 × 10^9^ cells per flask (assuming OD_600nm_ 1 = 10^9^ cells/mL in LB). In one of the two Erlenmeyer flasks, MMC was added (0.0125 µg/mL). After 3 h of incubation at 30°C with shaking, the entire suspension was filtered using GF/F filters. The filtrate was recovered in vials and stored at −20°C until measurement. All materials used, including vials, filter holders, falcon tubes, forceps, syringes, Erlenmeyer flasks, were acid-washed with HCl at a final concentration of 3.5%.

A few hours before measurements, samples were defrosted at 4°C in the dark. Dissolved organic matter (DOM) composition and size fractionation analysis were performed in filtrates using size exclusion chromatography with multi-detectors (DOC-LABOR) according to the methodology described in (52) and (53). Concentrations of bulk DOC, urea, ammonium, and DOC content in the operationally defined fractions—hydrophobic DOC (non-eluted), HMW (>10 kDa), “humic-like,” and LMW compounds (<0.5 kDA)—were quantified.

### Statistical analyses

All statistical analyses were performed using GraphPad Prism (version 8.0.1). Tests with a *P-*value < 0.05 were considered significant.

## Data Availability

The RNA-seq data obtained in this study are available through NCBI under BioProject PRJNA1133960.

## SUPPLEMENTAL MATERIAL

Supplemental figures aem.01564-25-s0001.pdf Figures S1 to S7.

Table S1 aem.01564-25-s0002.docx Strains used in this study.

Table S2 aem.01564-25-s0003.docx Plasmids used in this study.

Table S3 aem.01564-25-s0004.docx Primers used in this study.

Table S4 aem.01564-25-s0005.xlsx Complete list of the differentially expressed genes in the two pairwise comparisons.

Table S5 aem.01564-25-s0006.xlsx Prophage gene annotations and differential gene expression.

Table S6 aem.01564-25-s0007.xlsx Carbon and nitrogen sources released by *V. diazotrophicus*.

Video S1 aem.01564-25-s0008.avi Cell division of *V. diazotrophicus* wild type upon MMC induction.

Video S2 aem.01564-25-s0009.avi Cell division of prophage-free *V. diazotrophicus* mutant upon MMC induction.

ASM does not own the copyrights to Supplemental Material that may be linked to, or accessed through, an article. The authors have granted ASM a non-exclusive, world-wide license to publish the Supplemental Material files. Please contact the corresponding author directly for reuse.
