# Supplementary material for: The interplay between the marine diazotroph Vibrio diazotrophicus and its prophage shapes both biofilm structure and nitrogen release
Source: Appl Environ Microbiol. 2025 Dec 22;92(1):e01564-25. doi: 10.1128/aem.01564-25 (PMC12838380; doi:10.1128/aem.01564-25)
Supplement: Supplemental figures — Figures S1 to S7. [file aem.01564-25-s0001.pdf]

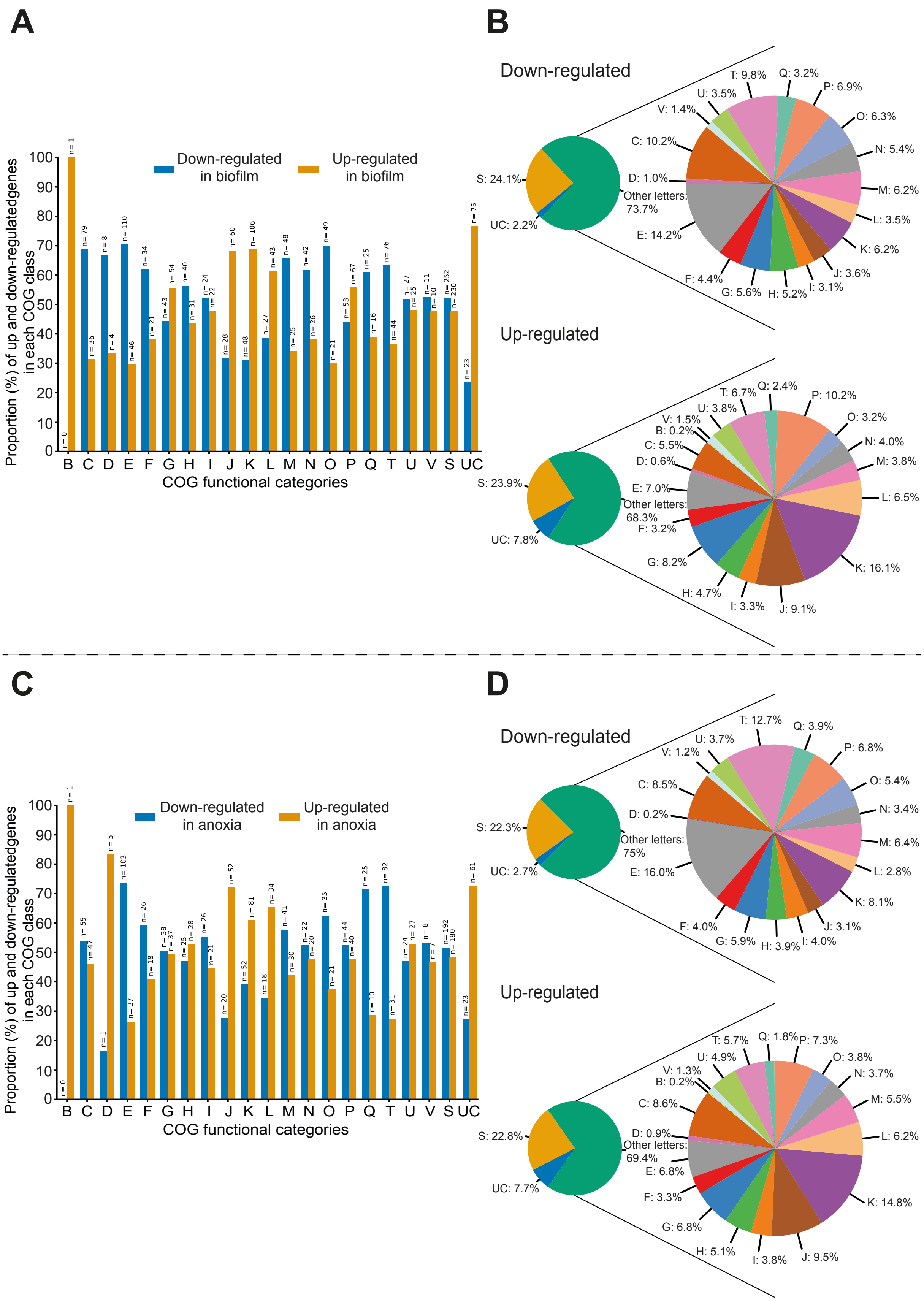
A
B
Proportion (%) of up and down-regulatedgenes in each COG class
Down-regulated in biofilm
Up-regulated in biofilm
COG functional categories
B C D E F G H I J K L M N O P Q T U V S UC
Down-regulated
S: 24.1%
UC: 2.2%
Other letters: 73.7%
T: 9.8%
Q: 3.2%
P: 6.9%
O: 6.3%
N: 5.4%
M: 6.2%
L: 3.5%
K: 6.2%
J: 3.6%
I: 3.1%
H: 5.2%
G: 5.6%
F: 4.4%
E: 14.2%
D: 1.0%
C: 10.2%
V: 1.4%
U: 3.5%
Up-regulated
S: 23.9%
UC: 7.8%
Other letters: 68.3%
T: 6.7%
Q: 2.4%
P: 10.2%
O: 3.2%
N: 4.0%
M: 3.8%
L: 6.5%
K: 16.1%
J: 9.1%
I: 3.3%
H: 4.7%
G: 8.2%
F: 3.2%
E: 7.0%
D: 0.6%
C: 5.5%
B: 0.2%
V: 1.5%
U: 3.8%
C
D
Proportion (%) of up and down-regulatedgenes in each COG class
Down-regulated in anoxia
Up-regulated in anoxia
COG functional categories
B C D E F G H I J K L M N O P Q T U V S UC
Down-regulated
S: 22.3%
UC: 2.7%
Other letters: 75%
T: 12.7%
Q: 3.9%
P: 6.8%
O: 5.4%
N: 3.4%
M: 6.4%
L: 2.8%
K: 8.1%
J: 3.1%
I: 4.0%
H: 3.9%
G: 5.9%
F: 4.0%
E: 16.0%
D: 0.2%
C: 8.5%
V: 1.2%
U: 3.7%
Up-regulated
S: 22.8%
UC: 7.7%
Other letters: 69.4%
T: 5.7%
Q: 1.8%
P: 7.3%
O: 3.8%
N: 3.7%
M: 5.5%
L: 6.2%
K: 14.8%
J: 9.5%
I: 3.8%
H: 5.1%
G: 6.8%
F: 3.3%
E: 6.8%
D: 0.9%
C: 8.6%
B: 0.2%
V: 1.3%
U: 4.9%

Fig. S1. COG analysis of the differentially expressed genes of *V. diazotrophicus* NS1, comparing cells grown in MDV versus MDV in biofilm (A, B) and MDV in anoxia (C, D). "n" mentioned above each bar represents the number of genes representing the bar.

COG letters correspond to A: RNA processing and modification. C: Energy production and conversion. D: Cell cycle control, cell division and chromosome distribution. E: Amino acid transport and metabolism. F: Nucleotide transport and metabolism. G: Carbohydrate transport and metabolism. H: Transport and metabolism of coenzymes. I: Lipid transport and metabolism. J: Ribosome translation, structure and biogenesis. K: Transcription. L: Replication, recombination and repair. M: Cell wall, membrane and envelope biogenesis. N: Cell motility. O: Post-translational modification, protein turnover and chaperones. P: Transport and metabolism of inorganic ions. Q: Biosynthesis, transport and catabolism of secondary metabolites. S: Function unknown. T: Signal transduction mechanisms. U: Intracellular traffic, secretion and vesicular transport. UC: Unknown genes. V: Defence mechanisms.

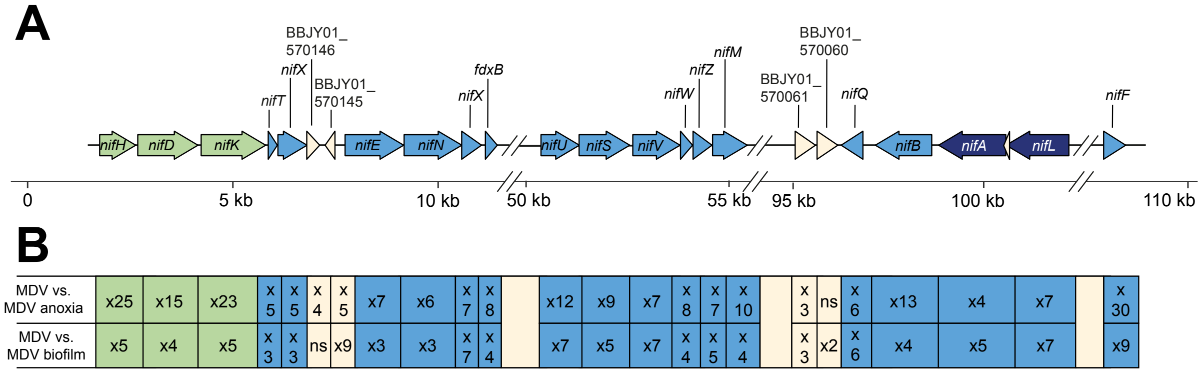

Fig. S2. Genomic structure of the *nif* cluster (A) and FoldChange of the different genes, comparing MDV with MDV in anoxia or in biofilm (B)

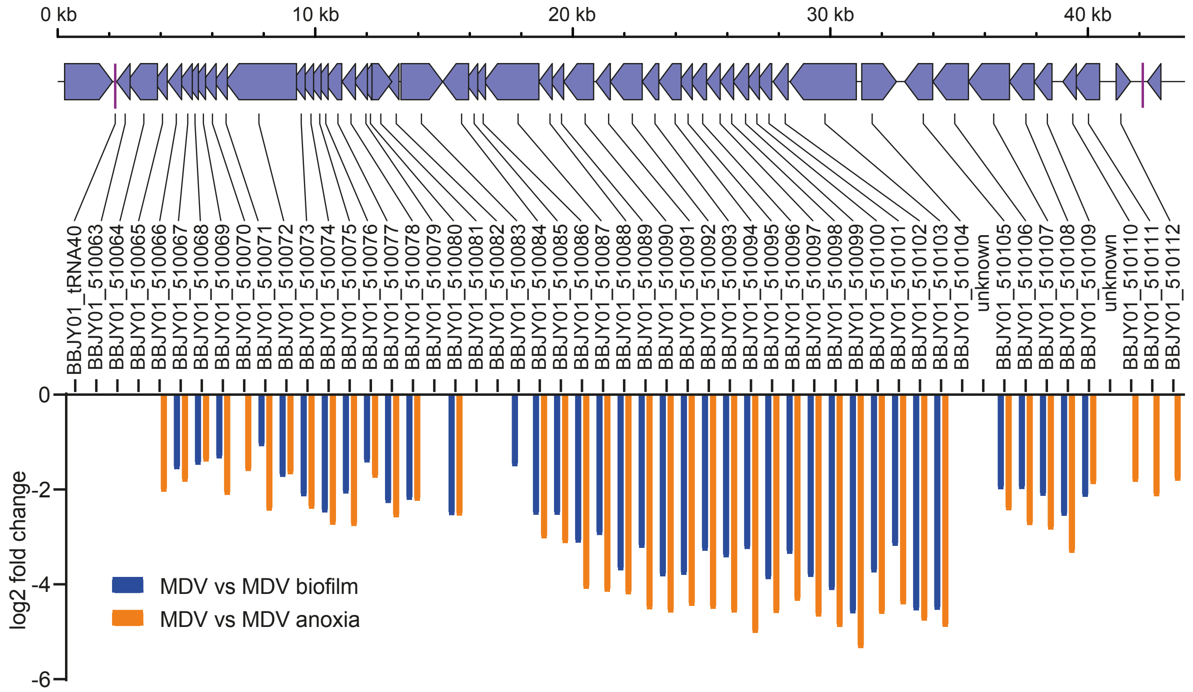

Fig. S3. Log2 FoldChange of the genes of Vdi_1 obtained when comparing MDV with MDV in biofilm or in anoxia.

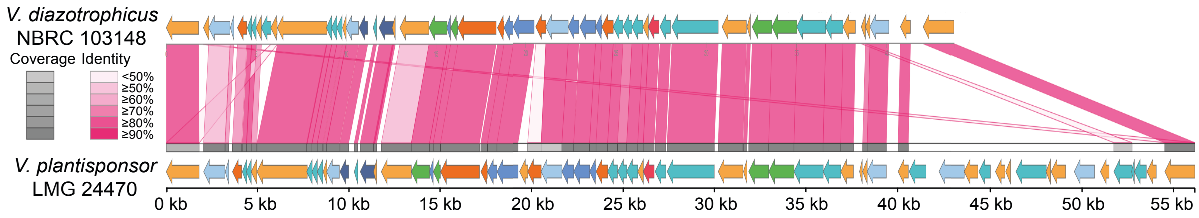

Fig. S4. Alignment of the genomic region of Vdi_1 with its closest relative, found in *V. plantisponsor* LMG24470. The alignment was done using PhageScope (1)

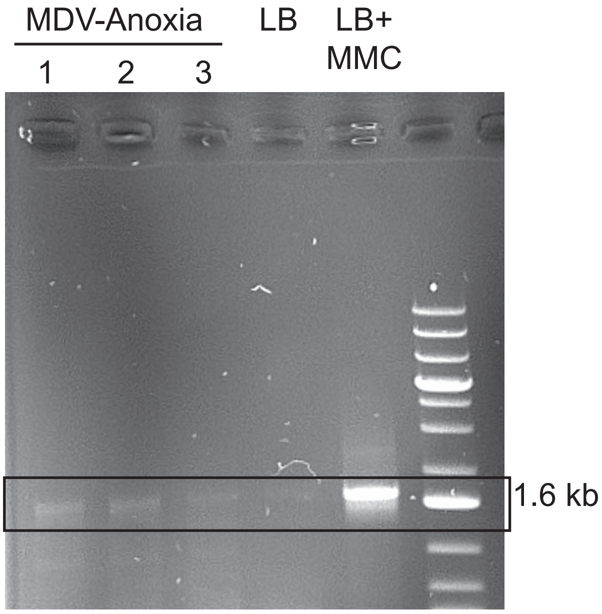

Fig. S5. Spontaneous excision of Vdi_1 from the genome of *V. diazotrophicus* NS1. A PCR was performed, using primers flanking the Vdi_1 insertion sites. If excised, a band at 1.6 kb is visible. See the bands at the expected size upon grown in liquid MDV under anoxic conditions.

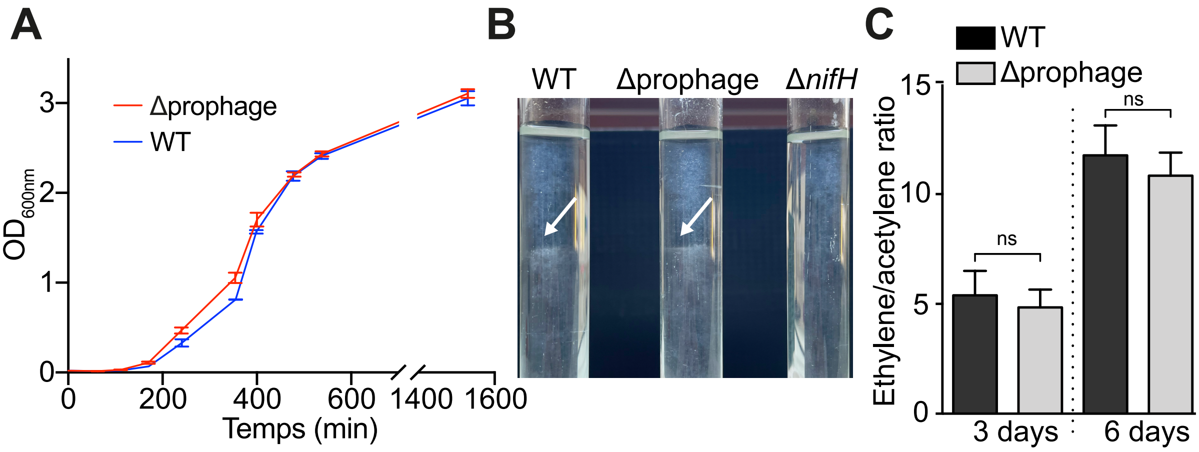

Fig. S6. Physiological experiments on *V. diazotrophicus* SN1 and the prophage-free mutant. (A) Growth curves of both strains when grown in LB. (B) Growth with N_2 as sole nitrogen source in softgellan. The *nifH* mutant corresponds to a previously constructed mutant (2), unable to grow in this condition. (C) ARA experiment on both strains after 3 and 6 days. Unpaired two-tailed t-tests were performed. ns=not significant.

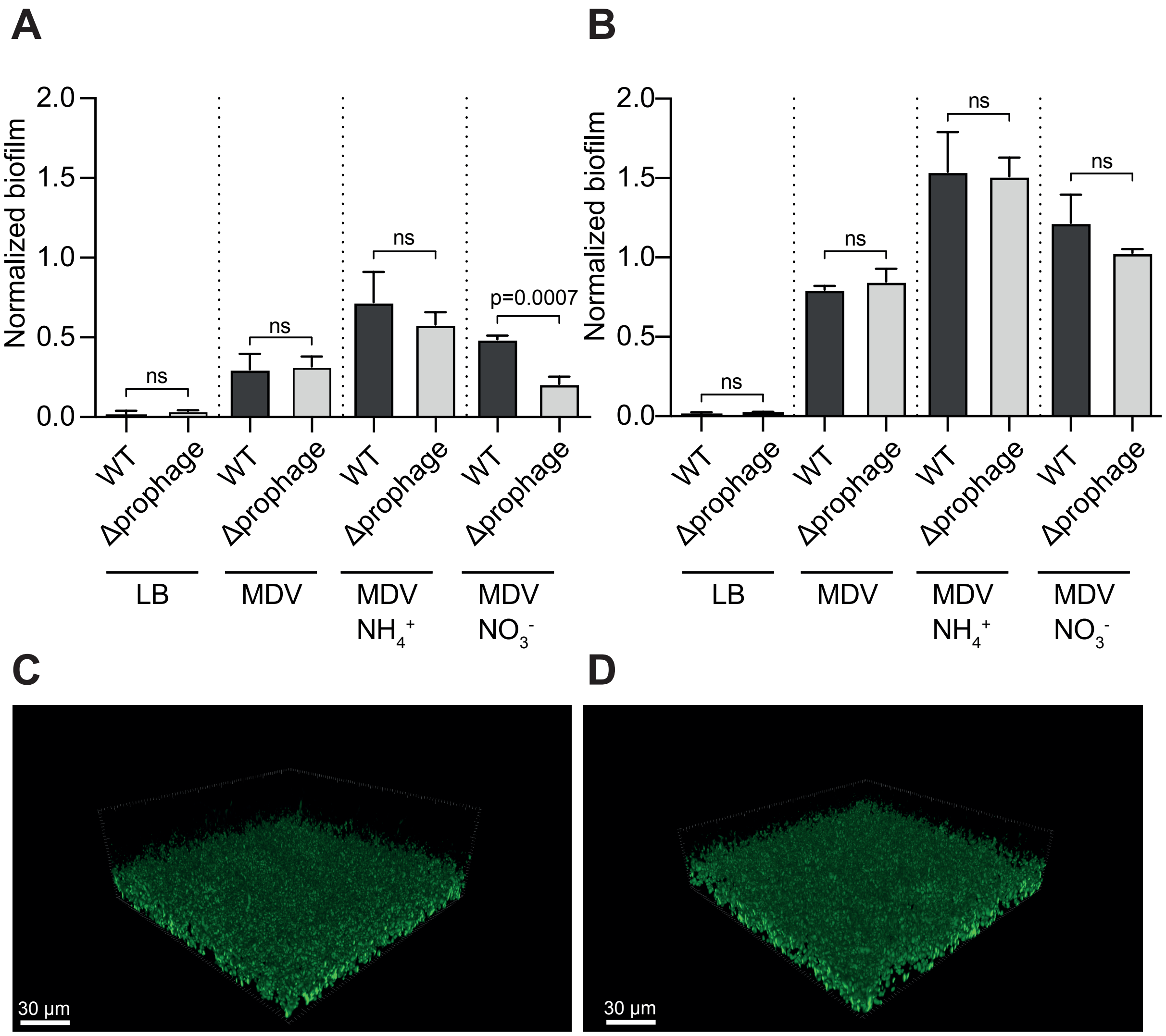

Fig. S7. Biofilm production by *V. diazotrophicus* NS1 or the prophage-free mutant under oxic conditions. Both strains were grown for 24 hours (A) or 48 hours (B) in microplates, under oxic conditions, in different media. Biofilm production was normalized by dividing OD_{550nm} by the OD_{600nm} obtained after 24 (A) or 48 h (B). Unpaired two-tailed t-tests were performed. ns=not significant. Stacked microscopy image showing the thickness of the biofilm produced by *V diazotrophicus* (C) or the prophage-free mutant (D) after 24 h of growth.

Supplementary movie S1. Timelapse movie of *V. diazotrophicus* NS1 upon induction with 0.0125 µg/ml MMC

Supplementary movie S2. Timelapse movie of the prophage-free mutant of *V. diazotrophicus* NS1 upon induction with 0.0125 µg/ml MMC

1. Wang RH, Yang S, Liu Z, Zhang Y, Wang X, Xu Z, Wang J, Li SC. 2024. PhageScope: a well-annotated bacteriophage database with automatic analyses and visualizations. Nucleic Acids Res 52:D756-D761.
2. Joublin-Delavat A, Touahri K, Cretin P, Morot A, Rodrigues S, Jesus B, Trigodet F, Delavat F. 2022. Genetic and physiological insights into the diazotrophic activity of a non-cyanobacterial marine diazotroph. Environ Microbiol 24:6510-6523.
