# Supplementary material for: The interplay between the marine diazotroph Vibrio diazotrophicus and its prophage shapes both biofilm structure and nitrogen release
Source: Appl Environ Microbiol. 2025 Dec 22;92(1):e01564-25. doi: 10.1128/aem.01564-25 (PMC12838380; doi:10.1128/aem.01564-25)
Supplement: Table S1 — Strains used in this study. [file aem.01564-25-s0002.docx]

| Strain collection number (US2B lab collection) | Strain name | Characteristics | References |
| --- | --- | --- | --- |
| *Escherichia coli* | | | |
| 204 | DH5α λpir | *sup E44, ΔlacU169 (ΦlacZΔM15), recA1, endA1, hsdR17, thi-1, gyrA96, relA1, λpir phage lysogen* | Lab collection |
| 206 | GEB883 + pEVS104 | Strain GEB883 (1) containing pEVS104 (2) | (3) |
| 207 | β-3914 | F^-^, RP4-2-Tc::Mu, Δ*dapA*::(*erm-pir*)  *gyrA462 zei-298::*Tn*10*, Km^R^, Em^R^, Tc^R^, DAP^-^ | (4) |
| *Vibrio diazotrophicus* | | | |
| 295 | *V. diazotrophicus* NBRC 103148 | Strain isolated from urchin gut | (5) |
| 301 | *V. diazotrophicus* pFD086 | Derivative of 295 containing pFD085 | (3) |
| 588 | *V. diazotrophicus* Δ*prophage* | Derivative of strain 295 in which the 40kb-prophage region has been deleted | This study |
| 594 | *V. diazotrophicus* Δ*prophage* pFD086 | Derivative of strain 588 containing pFD086 | This study |
| 595 | *V. diazotrophicus* Δ*prophage* pFD160 | Derivative of strain 588 containing pFD160 | This study |

Table S1. Strains used in this study.

1. Nguyen AN, Disconzi E, Charriere GM, Destoumieux-Garzon D, Bouloc P, Le Roux F, Jacq A. 2018. *csrB* gene duplication drives the evolution of redundant regulatory pathways controlling expression of the major toxic secreted metalloproteases in *Vibrio tasmaniensis* LGP32. mSphere 3.

2. Stabb EV, Ruby EG. 2002. RP4-based plasmids for conjugation between *Escherichia coli* and members of the *Vibrionaceae*. Bacterial Pathogenesis, Pt C 358:413-426.

3. Morot A, El Fekih S, Bidault A, Le Ferrand A, Jouault A, Kavousi J, Bazire A, Pichereau V, Dufour A, Paillard C, Delavat F. 2021. Virulence of *Vibrio harveyi* ORM4 towards the European abalone *Haliotis tuberculata* involves both quorum sensing and a type III secretion system. Environ Microbiol 23:5273-5288.

4. Le Roux F, Binesse J, Saulnier D, Mazel D. 2007. Construction of a *Vibrio splendidus* mutant lacking the metalloprotease gene *vsm* by use of a novel counterselectable suicide vector. Appl Environ Microbiol 73:777-84.

5. Guerinot ML, Patriquin DG. 1981. N_2_-fixing vibrios isolated from the gastrointestinal tract of sea urchins. Can J Microbiol 27:311-7.
