# Supplementary material for: The interplay between the marine diazotroph Vibrio diazotrophicus and its prophage shapes both biofilm structure and nitrogen release
Source: Appl Environ Microbiol. 2025 Dec 22;92(1):e01564-25. doi: 10.1128/aem.01564-25 (PMC12838380; doi:10.1128/aem.01564-25)
Supplement: Table S2 — Plasmids used in this study. [file aem.01564-25-s0003.docx]

| Plasmid name | Plasmid characteristics | Reference |
| --- | --- | --- |
| pGEM-T | Cloning vector. *lacZ*. Amp^R^ | Promega |
| pLP12 | Suicide plasmid used for targeted deletion of chromosomal genes of *Vibrio* species. *oriT*_RP4_, *oriV*_R6K_, P*_BAD_*-*vmi480*. Cm^R^ | (1) |
| pEVS104 | Conjugative helper plasmid. oriV_R6K_ oriT_RP4_. Km^R^ | (2) |
| pFD085 | Replicative plasmid for *Vibrio*, containing the P*_lac_* promoter upstream of the *gfp* gene of pFD085. Trim^R^ | (3) |
| pFD156 | Derivative of pLP12 containing the upstream and downstream fragments of the prophage region of *V. diazotrophicus*. Insertion performed using *Xma*I + *Eco*RI | This study |
| pFD160 | Derivative of pFD086 in which the P*_lac_* promoter has been replaced by the P*_510103_* promoter of *V. diazotrophicus*, using *Sal*I + *Sph*I | This study |

Table S2. Plasmids used in this study. All *in silico* plasmid sequences and maps are available upon request

1. Luo P, He X, Liu Q, Hu C. 2015. Developing universal genetic tools for rapid and efficient deletion mutation in *Vibrio* species based on suicide T-vectors carrying a novel counterselectable marker, Vmi480. PLoS One 10:e0144465.

2. Stabb EV, Ruby EG. 2002. RP4-based plasmids for conjugation between *Escherichia coli* and members of the *Vibrionaceae*. Bacterial Pathogenesis, Pt C 358:413-426.

3. Morot A, El Fekih S, Bidault A, Le Ferrand A, Jouault A, Kavousi J, Bazire A, Pichereau V, Dufour A, Paillard C, Delavat F. 2021. Virulence of *Vibrio harveyi* ORM4 towards the European abalone *Haliotis tuberculata* involves both quorum sensing and a type III secretion system. Environ Microbiol 23:5273-5288.
