# Supplementary material for: The interplay between the marine diazotroph Vibrio diazotrophicus and its prophage shapes both biofilm structure and nitrogen release
Source: Appl Environ Microbiol. 2025 Dec 22;92(1):e01564-25. doi: 10.1128/aem.01564-25 (PMC12838380; doi:10.1128/aem.01564-25)
Supplement: Table S3 — Primers used in this study. [file aem.01564-25-s0004.docx]

| Primer number | Primer name | Primer sequence | Target |
| --- | --- | --- | --- |
| 240213 | up-prophage-XmaI-F | ATGCCCCGGGcgacagcgcggcatcaaatg | Primer annealing around 800 bp upstream of the prophage region of *V. diazotrophicus* (strain 295), facing inwards. Used in combination with 240214, 240215 and 240216 to amplify the up-dn fragment of the prophage region, creating pFD156 |
| 240214 | up-prophage-R | ggcgtaaaaaagcccgtaac | Primer annealing at the beginning of the prophage region of *V. diazotrophicus* (strain 295), facing outwards. Used in combination with 240213, 240215 and 240216 to amplify the up-dn fragment of the prophage region, creating pFD156 |
| 240215 | dn-prophage-rcup-F | gttacgggcttttttacgccgctacaggctttttcgttgac | Primer annealing at the end of the prophage region of *V. diazotrophicus* (strain 295), facing outwards. Used in combination with 240213, 240214 and 240216 to amplify the up-dn fragment of the prophage region, creating pFD156 |
| 240216 | dn-prophage-EcoRI-R | ATGCGAATTCggcaatgacgcagtatattg | Primer annealing around 800 bp downstream of the prophage region of *V. diazotrophicus* (strain 295), facing inwards. Used in combination with 240213, 240214 and 240215 to amplify the up-dn fragment of the prophage region, creating pFD156 |
| 240217 | del-prophage-F | cgctggtctacttgggttg | Primer annealing within BBJY01_510062, facing towards the prophage region |
| 240218 | del-prophage-R | gcgctacgcaagcaaatttc | Primer annealing within BBJY01_510113, facing towards the prophage region |
| 240228 | P103-105-SalI-F | GATCGTCGACgtgaggtgagacttaatcatg | Primer annealing around the ATG region of gene BBJY01_510103, facing outwards |
| 240230 | P103-105-SphI-R | cgtgaacatcgaaagtaaatg | Primer annealing within gene BBJY01_510104, facing towards the ATG of BBJY01_510103. |

Table S3. Primers used in this study
